# Potential 14-3-3 binding sites in sirtuins reveal extended phosphosite-recognition modes

**DOI:** 10.1107/S2053230X25010908

**Published:** 2026-01-01

**Authors:** Michael Weyand, Laura Quast, Clemens Steegborn

**Affiliations:** a Department of Biochemistry University of Bayreuth Universitätsstrasse 30 95440Bayreuth Germany; University of Leipzig, Germany

**Keywords:** 14-3-3 proteins, sirtuins, phosphopeptides, regulation, protein–protein interaction

## Abstract

The adapter proteins of the 14-3-3 family modulate the activity and/or localization of their binding partners, which they capture if a generic target motif is in its phosphorylated state. Here, we report the identification of potential 14-3-3 binding sites in human sirtuin deacylases by bioinformatic analysis. We then characterize the interactions of peptides representing phosphorylation sites in sirtuin 3 (pS103) and sirtuin 1 (pS670) with 14-3-3 proteins. We further describe the crystal structures of complexes of 14-3-3σ with either of the two phosphopeptides. As a conclusion, we propose a more extended 14-3-3 binding mode on the N-terminal side of the phosphorylation site and the possibility of nongeneric motifs and conformations on the C-terminal side, still resulting in the known high binding affinity of the two partners.

## Abstract 2

Identification, binding measurements and structure determination of human sirtuin phosphopeptides in complex with human adapter protein 14-3-3σ reveal variations among tight binders and suggest extended binding modes.

## 1. Introduction

The 14-3-3 family of adapter proteins modulate the activity and/or localization of their binding partners (Kleppe *et al.*, 2011 ▸). They recognize a Ser/Thr phosphorylation in a short sequence motif that is generally assumed to belong to either of two sequence patterns, also referred to as ‘binding modes’ I and II, or a carboxy-terminal ‘mode III’ motif (Aitken, 2006 ▸). At least 133 well characterized physiological 14-3-3 binding sites distributed across 98 different proteins have been reported (Aitken, 2006 ▸). The short, ∼10-residue recognition sites insert into a groove of the conserved, W-shaped architecture of dimeric 14-3-3 (Aitken, 1996 ▸; Obsil & Obsilova, 2011 ▸). Today, crystal structures of all seven mammalian apo 14-3-3 isoforms are available, together with several synthetic phosphopeptide mode 1 and 2 complexes. For example, complexes with peptides derived from Raf (Petosa *et al.*, 1998 ▸; Kaplan *et al.*, 2020 ▸), integrin α4 (Bonet *et al.*, 2013 ▸), RapGef2 (Kaplan *et al.*, 2020 ▸) or protein kinase R (Andlovic *et al.*, 2023 ▸) have been determined. Also, structures of 14-3-3 proteins in complex with complete phosphorylated partner proteins have been reported, initially with serotonin *N*-acetyltransferase (Obsil *et al.*, 2001 ▸) and subequently with neutral trehalase Nth1 (Alblova *et al.*, 2017 ▸) and also with B-Raf (Martinez Fiesco *et al.* (2022 ▸). These structures confirmed that the binding mode of peptide ligands by 14-3-3 proteins is equivalent to their interactions with complete protein ligands (Obsilova & Obsil, 2022 ▸). 14-3-3 binding in most cases leads to a 1:1 complex with a single target protein, with each 14-3-3 monomer binding to an identical target sequence site and the 14-3-3 dimer thus recruiting two monomers of the target. However, the 14-3-3 dimer can also bind two different targets (Zha *et al.*, 1996 ▸), or a single protein target at two distinct phosphorylation sites (for example histone H3; Winter *et al.*, 2008 ▸). In the latter case, only a double phosphorylation leads to a ∼100-fold higher affinity, with a *K*_d_ of about ∼5–10 µ*M*, compared to a single phosphorylated histone protein. Lower *K*_d_ values could also be observed for 14-3-3 isoforms β, η, τ and ζ in the case of the pS-Raf-259 peptide, with values of ∼120–145 n*M* (Muslin *et al.*, 1996 ▸).

The 14-3-3 proteins also appear to interact with members of the sirtuin protein family. Sirtuins form the conserved protein deacylase (PDAC) class III and regulate metabolism and aging processes. In mammals, seven sirtuin isoforms (Sirt1–Sirt7) mediate adaptive responses to a variety of stresses, including calorie restriction and metabolic stress. Sirt1, Sirt6 and Sirt7 are mainly located in the nucleus, Sirt3–Sirt5 in mitochondria and Sirt2 in the cytoplasm. Sirt1 has been implicated, for example, in the control of chromatin structure, genomic stability and energy metabolism. Sirt1 targets include the transcription factors p53 and FOXO3a and the DNA-repair proteins Ku70 and XPA. The mitochondrial Sirt3 regulates the acetylation levels of a wide variety of metabolic enzymes, such as acetyl coenzyme A synthetase 2, hydroxymethylglutaryl-CoA synthase 2 and proteins of the electron-transfer chain (Hirschey *et al.*, 2010 ▸; Shimazu *et al.*, 2010 ▸; Rardin *et al.*, 2013 ▸). Sirt3 thereby regulates oxidative phosphorylation (Cimen *et al.*, 2010 ▸; Finley *et al.*, 2011 ▸), ketogenesis and oxidative burst responses (Tao *et al.*, 2010 ▸; Chen *et al.*, 2011 ▸).

A 14-3-3-dependent regulation of sirtuin function is still based on a limited number of publications. The first 14-3-3–sirtuin cross-talk was suggested by findings that the *Caeno­rhabditis elegans* SIR2 ortholog SIR-2.1 (mammalian Sirt1) binds to both of the worm 14-3-3 proteins PAR-5 and FFT-2 (Berdichevsky *et al.*, 2006 ▸; Wang *et al.*, 2006 ▸), regulating the interaction of the deacylase with a transcription factor upon stress signals. A direct interaction with Sirt2 was also reported for mammalian 14-3-3 isoforms β and γ, causing a down-regulation of p53 activity (Jin *et al.*, 2008 ▸). In yeast, simultaneous binding of the 14-3-3 protein Bmh1 to phosphorylated histone H3 and the phosphorylated sirtuin Hst2 was observed (Jain *et al.*, 2021 ▸). Interestingly, the human Sirt2 residue corresponding to the phosphorylated serine in Hst2 was reported to be phosphorylated during Sirt2 chromatin recruitment (Movahedi Naini *et al.*, 2015 ▸; Pereira *et al.*, 2018 ▸). In human cells, 14-3-3ζ was reported to regulate a Sirt1-dependent skin-repair mechanism (Wu *et al.*, 2021 ▸).

In a reverse signaling mode, 14-3-3 proteins might also be targets or downstream effectors of sirtuins. A sirtuin appears to regulate anoxia–reoxygenation tolerance via regulation of 14-3-3ζ and BAD binding (Lynn *et al.*, 2008 ▸). In a biotin-based proteomic approach the same 14-3-3 isoform was identified as a target of Sirt1 for caspase-2 regulation (Andersen *et al.*, 2011 ▸). The finding that 14-3-3 proteins can regulate sirtuins, and vice versa, identifies their interactions as a complex signaling cross-talk interface.

To elucidate the molecular details of human sirtuin binding to 14-3-3 proteins, we first identified potential 14-3-3-responsive sirtuin phosphorylation sites through a position-specific scoring matrix (PSSM) sequence analysis (Thiel, 2015 ▸). The binding of corresponding phosphopeptides to 14-3-3 proteins was analyzed by microscale thermophoresis and isothermal titration calorimetry. Two of the identified sirtuin sites interacted with 14-3-3 proteins with low micromolar affinity. Crystal structure analysis revealed that the identified sequences, despite deviating from known interaction patterns, partly exploit conserved interaction modes and partly reveal new ways for tight binding, thereby expanding our knowledge of how targets interact with 14-3-3 proteins.

## 2. Materials and methods

Phosphorylated and nonphosphorylated sirtuin peptides were chemically synthesized by GL Biochem, Shanghai, China. All other chemicals were purchased at the highest purity grade from Sigma–Aldrich.

### 2.1. Protein expression and purification

#### 2.1.1. Cloning

For all seven human 14-3-3 isoforms, full-length (14-3-3-fl) and a C-terminally truncated (14-3-3-ΔC) gene fragments were cloned into pPROEX-HTb (GE Healthcare, Little Chalfont, United Kingdom) using BamHI and SalI restriction sites.

#### 2.1.2. Purification

The expression and purification of His_6_-tagged 14-3-3 proteins were carried out using standard procedures (Yang *et al.*, 2006 ▸). In brief, *Escherichia coli* BL21 Rosetta (DE3) cells (Merck, Darmstadt, Germany) were transformed with the pProExHTb vector (Invitrogen, Paisley, United Kingdom) carrying the respective 14-3-3 gene. The cells were grown in Terrific Broth medium at 37°C to a density of *A*_600_ = 0.6 and were induced with 0.4 m*M* isopropyl β-d-1-thiogalactopyranoside (IPTG). After IPTG addition, the cells were grown for a further 16 h at 20°C before harvesting. The cells were lysed in buffer *A* [50 m*M* Tris pH 8.0, 5%(*v*/*v*) glycerol, 2 m*M* β-mercapto­ethanol] which contained 300 m*M* NaCl, 10 m*M* imidazole and 1 m*M* phenylmethylsulfonyl fluoride using a microfluidizer. After centrifugation, the supernatant was applied onto a nickel–nitrilotriacetic acid column and washed with buffer *A* containing 500 m*M* NaCl and 25 m*M* imidazole. After elution with buffer *A* containing 300 m*M* NaCl and 250 m*M* imidazole, the protein was concentrated using a 30 000 molecular-weight cutoff Amicon ultrafiltration membrane (Millipore, Billerica, Massachusetts, USA). The protein concentration was determined using UV–visible spectrometry (NanoDrop, Thermo Scientific, Wilmington, Delaware, USA) and the purity was verified by SDS–PAGE. The protein was dialyzed against 20 m*M* HEPES–NaOH pH 7.5, 100 m*M* NaCl, 10 m*M* MgCl_2_, 1.0 m*M* TCEP and stored at −80°C.

### 2.2. Microscale thermophoresis (MST)

**Figure 1:**
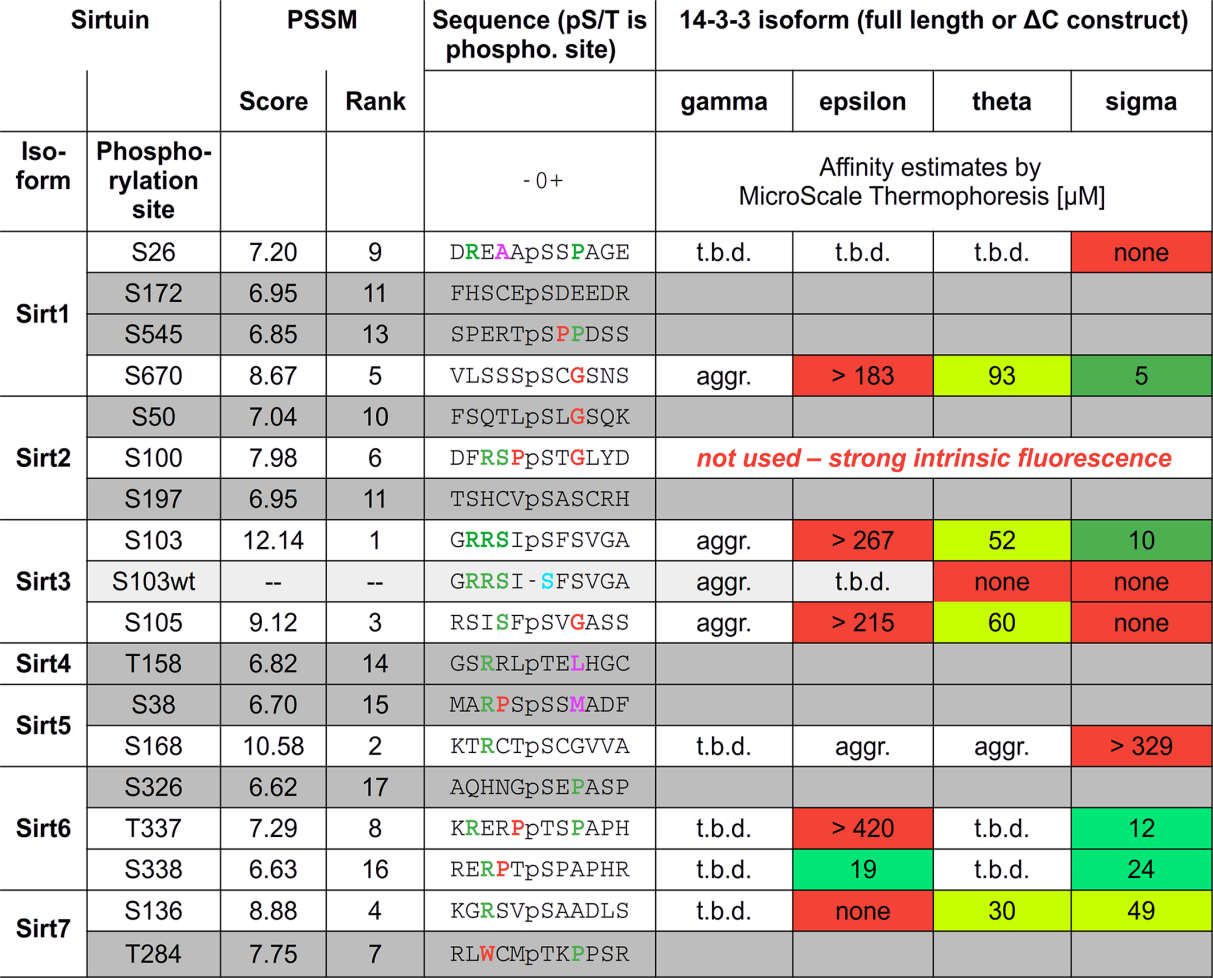
Classification and analysis of sirtuin PSSM results for potential 14-3-3 phosphoserine binding sites. Peptides with a gray background were not analyzed experimentally. Score is the overall PSSM score for the phosphorylation site; rank is the order within the 17 proposed sirtuin binding sites. Conserved residues in the 14-3-3 motifs are marked in green. Residues with restricted (proline) or very flexible (glycine) protein backbone or with a very large side chain (tryptophan) are marked in red. Amino acids with the same or similar hydrophobicity/size are shown in magenta. *K*_d_ values from duplicate measurements are colored green for *K*_d_ < 100 µ*M*. Fields marked in red indicate no binding or no successful experiment.

The binding of phosphorylated sirtuin peptides (Fig. 1 ▸) was analyzed using the change in the intrinsic fluorescence signal of the 14-3-3 proteins measured with label-free microscale thermophoresis using a Nanotemper Monolith NT.LabelFree or NT.115. A typical binding experiment using 0.6 µ*M* 14-3-3 protein was performed in a modified MST buffer consisting of 25 m*M* MES pH 6.5, 5 m*M* MgCl_2_, 5 m*M* CaCl_2_. The measurements were performed at 25°C using a LED power of 10–40%. All measurements were performed at least in duplicate, and the results shown are representative measurements individually fitted using *GraFit* 7 (Erathicus Software, Horley, United Kingdom).

### 2.3. Isothermal titration calorimetry (ITC)

Peptide ligands were freshly prepared in the exact same buffer as was used for the gel-filtration run. Samples were degassed and temperature-equilibrated using a degassing station (TA Instruments). A volume of 250 µl of 14-3-3σ-ΔC protein sample at a concentration of 150 µ*M* was transferred into the sample cell of a nanoITC (TA Instruments) and 50 µl of a fourfold-concentrated phosphorylated peptide was loaded into the injection needle. Multiple injection measurements were carried out at 25°C with a 300 rev min^−1^ stirring rate and 300 s spacing between each 2 µl injection. The heat quantity post-injection was determined by integration of the measured peaks. The Sirt1^pS670^ peptide behaved best and was measured in duplicate. Subtraction of heat of dilution measurements, peak integration and one-site binding fits were performed with *NanoAnalyze* (TA Instruments). The reported errors are the errors between fits of the measured duplicates.

### 2.4. Crystallization, structure determination and refinement

For crystallization of the 14-3-3σ–peptide complexes, protein and Sirt1^pS670^, Sirt3^pS103^, Sirt3^pS105^, Sirt6^pT337^ or Sirt6^pS338^ peptides were mixed in a 1:1.5 molar ratio in 20 m*M* HEPES–NaOH pH 7.5, 2 m*M* MgCl_2_, 1 m*M* TCEP and set up for crystallization in 0.1 *M* HEPES–NaOH pH 7.5, 0.2 *M* CaCl_2_, 24–28%(*v*/*v*) PEG 400, 5%(*v*/*v*) glycerol, 1 m*M* TCEP at 4°C (Table 1 ▸). Crystals of the Sirt1^pS670^ and Sirt3^pS103^ complexes grew within a week and could directly be flash-cooled in liquid nitrogen. No crystallization could be observed for either of the Sirt6 peptides.

Data collection was performed at BESSY (Berlin, Germany) on MX beamline BL14.1 (Mueller *et al.*, 2015 ▸), using a PILATUS 6M detector, at a wavelength of 0.91804 Å and a cryogenic temperature of 100 K, and the data were processed with *XDS* (Kabsch, 2010 ▸). Molecular replacement was carried out with *Phaser* (McCoy *et al.*, 2007 ▸) using the structure of 14-3-3σ (PDB entry 1ywt; Wilker *et al.*, 2005 ▸) as the search model. The obtained model was subjected to iterative rounds of model building and refinement using *Coot* (Emsley *et al.*, 2010 ▸) and *REFMAC* (Murshudov *et al.*, 2011 ▸). Data-collection and refinement statistics are given in Tables 2 ▸ and 3 ▸. All structural figures were prepared with *PyMOL* (version 2.5; Schrödinger).

## 3. Results and discussion

### 3.1. Sirtuin sequence alignment, PSSM analysis and *in vitro* characterizations

**Figure 2:**
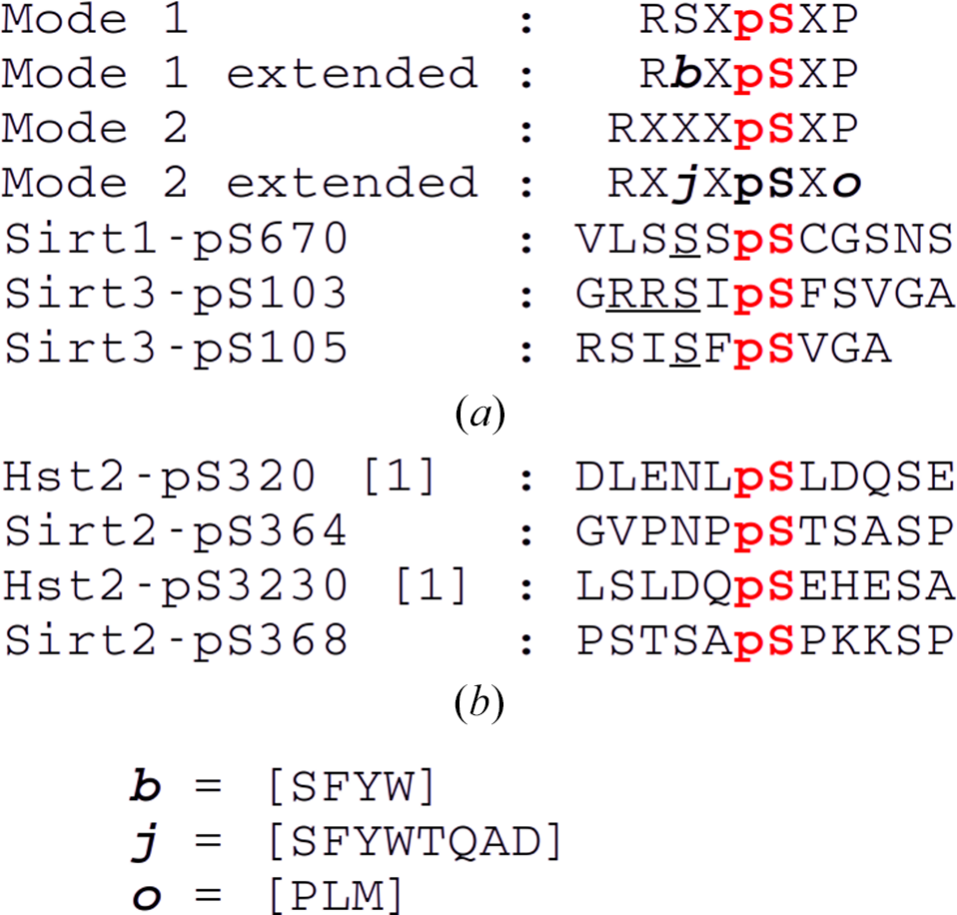
Sequence motifs for 14-3-3 binding. (*a*) Classical (Aitken, 1996 ▸) and extended (Yaffe *et al.*, 1997 ▸; Johnson *et al.*, 2010 ▸) 14-3-3 phospho-binding motifs and sirtuin 1 and 3 binding sequences. Sirtuin amino-acid positions that are conserved in mode 1 or 2 are underlined. (*b*) Sequence alignments of yeast Hst2 and human sirtuin 2 for the reported yeast–14-3-3 interaction sites (Jain *et al.*, 2021 ▸).

To study physiological interactions of human sirtuins with 14-3-3 adapter proteins, we performed a bioinformatics analysis for potential 14-3-3 binding sites in all seven human sirtuin isoforms. We used position-specific sequence matching (PSSM; Thiel, 2015 ▸) to identify 25 potential Ser and Thr phosphosites, including nine sites that were not described in previous work reporting such an analysis (Madeira *et al.*, 2015 ▸). Of these, 17 sites scored above the software significance threshold (Fig. 1 ▸), comprising sequences from all human sirtuin isoforms. All 17 sites were compared with database and literature information concerning their phosphorylation. Furthermore, all sites were compared with known 14-3-3 binding sites and the previously proposed binding motifs I and II (Fig. 2 ▸*a*; Yaffe *et al.*, 1997 ▸) through manual sequence comparison and by homology modeling based on high-resolution structures of 14-3-3–phosphopeptide complexes for both binding modes. For none of the PSSM-predicted sites could a confirmation of phosphorylation be identified from the literature and *PhosphoSitePlus* (version 6.8.1; Hornbeck *et al.*, 2015 ▸).

**Figure 3:**
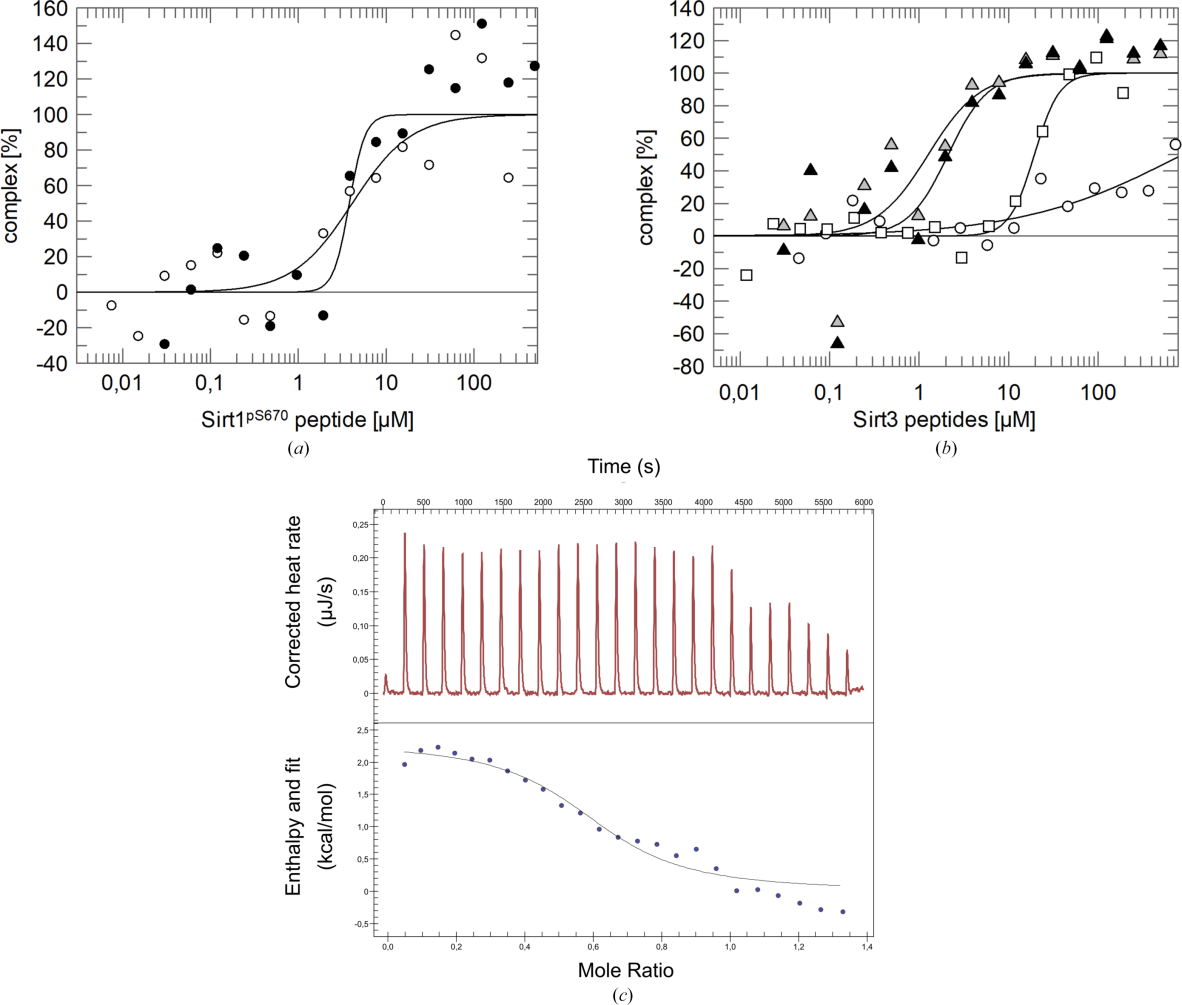
Affinity measurements of Sirt1^pS670^ and different Sirt3 peptides for 14-3-3σ-ΔC protein using MST and ITC experiments. (*a*) Two independent MST experiments of the titration of 14-3-3σ-ΔC with Sirt1^pS670^ (circles and filled circles, respectively), leading to an average *K*_d_ of 1.7 ± 1.3 µ*M*. (*b*) MST experiments of titrations of 14-3-3σ-ΔC with Sirt3^pS105^ (squares) or Sirt3^pS103^ (two independent runs, filled triangles and triangles) endecamer peptides and, for comparison, with nonphosphoylated Sirt3^S103^ peptide (circles). The phosphorylated peptides showed binding to 14-3-3σ-ΔC with *K*_d_ values of 1.5 ± 1.2 and 2.1 ± 1.1 µ*M*, respectively, for Sirt3^pS103^ and 19.4 ± 3.0 µ*M* for Sirt3^pS105^. In contrast, the nonphosphorylated peptide Sirt3^S103^ showed no significant affinity for 14-3-3σ-ΔC (∼881 ± 2148 µ*M*). (*c*) ITC measurement of the titration of 14-3-3-ΔC protein with Sirt1^pS670^ phospho-endecamer peptide yielded an apparent *K*_d_ of 5.0 ± 4.2 µ*M*, in good agreement with the MST results.

For nine of the sites, including sites from all sirtuin isoforms except Sirt4, endecameric phosphopeptides were chemically synthesized for *in vitro* analysis. Microscale thermophoresis binding measurements were initially performed with the truncated 14-3-3σ-ΔC protein due to its known high temperature stability and best crystallization behavior among 14-3-3 isoforms. Of the seven phosphopeptides tested, three showed no or very weak binding to 14-3-3σ (Figs. 1 ▸, 3 ▸*a* and 3 ▸*b*). The best binders to 14-3-3σ were the peptides representing Sirt6^pT336^, Sirt6^pS338^, Sirt3^pS103^, Sirt3^pS105^ and, surprisingly, Sirt1^pS670^, which shows no or very low sequence similarity to both 14-3-3 binding motifs (Figs. 1 ▸ and 2 ▸*a*). All *K*_d_ values are in the lower two-digit micromolar range and thus are comparable to previously reported values for 14-3-3–target interactions (Wang *et al.*, 1999 ▸; Gogl *et al.*, 2021 ▸). For the Sirt1 peptide, a similar *K*_d_ value was obtained from microscale calorimetry experiments (Fig. 3 ▸*c*).

Testing a nonphosphorylated version of the Sirt3-103 sequence site peptide, Sirt3^S103^, showed no significant binding affinity for 14-3-3σ, confirming the relevance of the modification and thus the generic, phosphorylation-dependent binding of a 14-3-3 ligand (Fig. 3 ▸). A comparison of the sequences of the Sirt3-103 and Sirt3-105 sites further illustrates the relevance of the target sequence around the phosphorylation site for both recognition by 14-3-3 proteins in general and for 14-3-3 isoform differences in target selection. While Sirt3^pS103^ binds tightly to 14-3-3σ, the Sirt3^pS105^ peptide shows no affinity. This result is in agreement with 14-3-3 specificity data from the analysis of a peptide libraray (Yaffe *et al.*, 1997 ▸), which indicated an RRSRpSYP*XX* optimum sequence for specific 14-3-3σ binding. In contrast to the Sirt3-103 site, the Sirt3-105 sequence motif lacks the conserved +2 proline and also the positive RR*X*{R/H/K} patch on the N-terminal side of the phosphorylation site. In contrast, the tight and 14-3-3σ-specific binding that we observed for the Sirt1^pS670^ peptide, which lacks all of the position criteria of the previous study (Yaffe *et al.*, 1997 ▸), contradicts this optimum sequence and again indicates a wider variation in recognition motifs than previously assumed.

### 3.2. Molecular interaction of sirtuin phosphosites with 14-3-3σ

**Figure 4:**
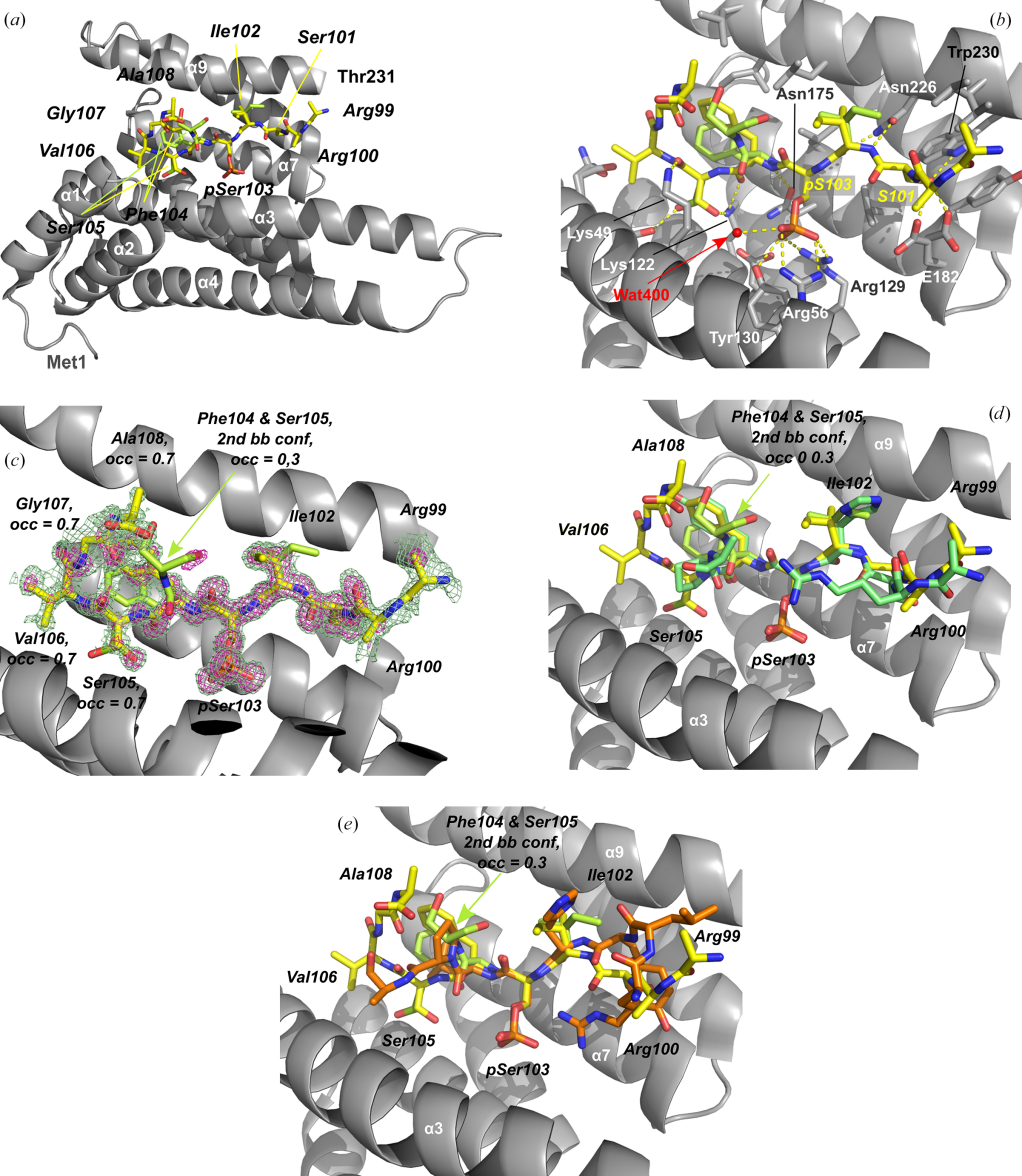
Crystal structure of the 14-3-3σ–Sirt3^pS103^ complex. (*a*) Content of the asymmetric unit showing a 14-3-3σ monomer with bound Sirt3 peptide. The normal physiological 14-3-3 dimer is formed by a symmetry-related molecule (see Supplementary Fig. S1). (*b*) Sirt3 peptide binding site with hydrogen-bonding network. (*c*) OMIT (by omitting Sirt3 peptide atoms) electron densities of the Sirt3 peptide contoured at 2σ (green) and at 4σ (magenta). (*d*, *e*) Structure superposition of Sirt3 peptide with (*d*) 14-3-3 binding mode 1 (PDB entry 1qjb) and (*e*) mode 2 (PDB entry 1qja) peptides by fitting the pSer residue using the *Coot* ‘superpose ligand’ function. C-atom coloring: Sirt3, peptide yellow; mode 1 peptide, green; mode 2 peptide, orange.

To understand the structural basis of 14-3-3 recognition of the nongeneric binding motifs we identified in human sirtuins, we performed crystallization experiments with the well crystallizing 14-3-3σ-ΔC construct and phosphopeptides representing sites in Sirt1, Sirt3 and Sirt6. For Sirt1^pS670^ and Sirt3^pS103^, crystals of their 14-3-3σ-ΔC complexes could be obtained and high-resolution diffraction data were collected on BESSY beamline BL14.1. The 14-3-3σ-ΔC complexes were solved by molecular replacement with PDB entry 1ywt as the search model (Figs. 4 ▸ and 5 ▸). The complex structures were refined for the Sirt3^pS103^ peptide at 1.11 Å resolution to *R*_cryst_ and *R*_free_ values of 12.8% and 14.5%, respectively, and for the Sirt1^pS670^ peptide at 1.64 Å resolution to *R*_cryst_ and *R*_free_ values of 17.6% and 20.2%, respectively (Table 3 ▸). The structures feature electron density for all 231 residues of the 14-3-3 monomer in the asymmetric unit, with a gap between Glu71 and Gly78 in the case of the Sirt1^pS670^ complex. Each 14-3-3 monomer has the established overall structure for this protein class, with nine antiparallel helices. The phosphopeptide complexes arrange with a symmetry mate into the classical ‘W’ form to build a large central binding cavity (Supplementary Fig. S1). For both phosphopeptides, a single molecule is well defined by electron density and occupies the central generic peptide-binding groove of 14-3-3σ-ΔC in an extended conformation.

In both peptide complexes, the negative charge of the phosphate moiety of the phosphoserine is compensated by the classical positively charged patch formed by Arg56 and Arg129, and a hydrogen bond to Tyr130. The Sirt1^pS670^ phosphopeptide complex also features the typical phosphate interaction with Lys49. In contrast, the Sirt3^pS103^ complex lacks this phosphate interaction with Lys49, which instead forms a hydrogen bond to the +2 (amino-acid sequence position relative to the phosphorylation site) carbonyl oxygen. Despite this small difference, both sirtuin phosphopeptides show the high-affinity *in vitro* binding of classical 14-3-3 binding motifs.

The most striking feature of the 14-3-3 binding motifs proposed for both sirtuins is the substitution of the assumed to be conserved Pro at the +2 position by a small hydrophilic residue or the flexible Gly. These residues also deviate from the Leu or Met substituting for Pro at this position in previously reported extended 14-3-3 binding modes (Johnson *et al.*, 2010 ▸). On the other side, both sirtuin modes contain a serine at the −2 position and, in the case of Sirt3, a conserved arginine residue at −3 (mode 1; at −4 in mode 2; Figs. 2 ▸*a* and 4 ▸). In contrast, the Sirt1 peptide lacks any sequence similarity to both classical 14-3-3 peptide modes except for the Ser at the −2 position. Still, both sirtuin peptides bind in a very similar way on the N-terminal side of pSer, ‘fixed’ by the only specific peptide side-chain hydrogen bond of this conserved Ser to Trp230 and Glu182 of the 14-3-3σ protein. Furthermore, the generic Arg residues in Sirt3 both show highly flexible side chains and in fact were excluded from the final model due to a lack of defining electron density. This observation agrees with the Arg conformations in the high-resolution 14-3-3 complex with mode 1 nuclear export signal phosphopeptide (PDB entry 1qjb; Wilker *et al.*, 2005 ▸). This complex consists of two 14-3-3 monomers in the asymmetric unit with two different peptide Arg side-chain conformations in each subunit in weak electron density and with high ADP values, also indicating disordered side chains for this conserved mode 1 residue. Our results thus fit to these previous observations and suggest that binding-motif variations N-terminal to the phosphosite can be accommodated without a significant loss in binding affinity. This observation is also consistent with the lack of tight binding for Sirt3^p105^, which lacks the Arg residues, and might indicate a primarily electrostatic contribution of this target region, depending on the charge distribution but not so much on the exact atom positions.

In the Sirt3^pS103^ complex, the peptide backbone shows a very similar conformation on the N-terminal side of the phosphate moiety as in the 14-3-3 mode 1 complexes. On the C-terminal side, it contains a sequence strongly deviating from previously identified interaction motifs and adopts a unique backbone conformation due to the loss of the +2 Pro. Rather than leaving the peptide-binding site, as seen in classical mode 1 complexes, the Sirt3 peptide backbone reorients the 14-3-3 Lys49 side chain, which forms a hydrogen bond to a Sirt3 peptide carbonyl instead of the normally observed interaction with the phosphate moiety. Interestingly, the Sirt3 peptide binds with (at least) two backbone conformations at this C-terminal side of the phosphorylated serine. The main conformation could be modeled with an occupancy of 70%. A second backbone conformation could only be modeled for Sirt3 residues Phe104 and Ser105 (Fig. 4 ▸). The crystal-packing strength of the Sirt3 peptide complex appears to be unaffected, as indicated by the very high diffraction (Table 2 ▸), which is in agreement with all 14-3-3 peptide-complex structures, but shows a so far unobserved heterogenous 14-3-3 peptide binding partner. This new Lys49 side-chain hydrogen-bond conformation provides a rationale for efficient ligand binding by 14-3-3 proteins in the absence of one of the classical binding motifs.

The Sirt1^pS670^ complex supports the model of structurally heterogenous yet tight binding on the C-terminal side of the phosphoserine suggested by the Sirt3 peptide complex. Compared with all previously reported 14-3-3 target sequences, the Sirt1 peptide (pSCGSNS) features the most flexible sequence. However, this high backbone flexibility is compensated by a new sequence feature of the Sirt1 motif: the small residues at the C-terminal side of the phosphorylated serine lead to a specific side-chain hydrogen bond from the +4 Asp to the carbonyl oxygen of 14-3-3σ Leu218. Such an interaction has so far not been observed in any 14-3-3 phosphopeptide complex. Our high-resolution diffraction data further allowed the positional refinement of multiple side-chain conformations for the phosphopeptide, which indicate that this sequence part of the 14-3-3 ligand either does not contribute significantly to the binding affinity and thus can tolerate larger sequence variations or can contribute through varying interactions. Our complex structures thus suggest a C-terminally extended (up to five amino acids upstream from the phosphorylation site) and more variable sequence motif for 14-3-3 binding. Although our data are restricted to peptide ligands, the respective motifs are located in sirtuin regions that are assumed to be flexible since all phosphorylation sites are located, according to the UniProt description, outside the structurally known (or assumed) domains. They are thus likely to reflect the interaction modes of the full-length proteins and can therefore serve as a basis for further cellular studies on 14-3-3 binding to sirtuins and other proteins via extended binding modes.

## Supplementary Material

PDB reference: 14-3-3σ–sirtuin 1 phosphopeptide complex, 8anb

PDB reference: 14-3-3σ–sirtuin 3 phosphopeptide complex, 8anc

Supplementary Figure S1. DOI: 10.1107/S2053230X25010908/no5215sup1.pdf

## Figures and Tables

**Figure 5:**
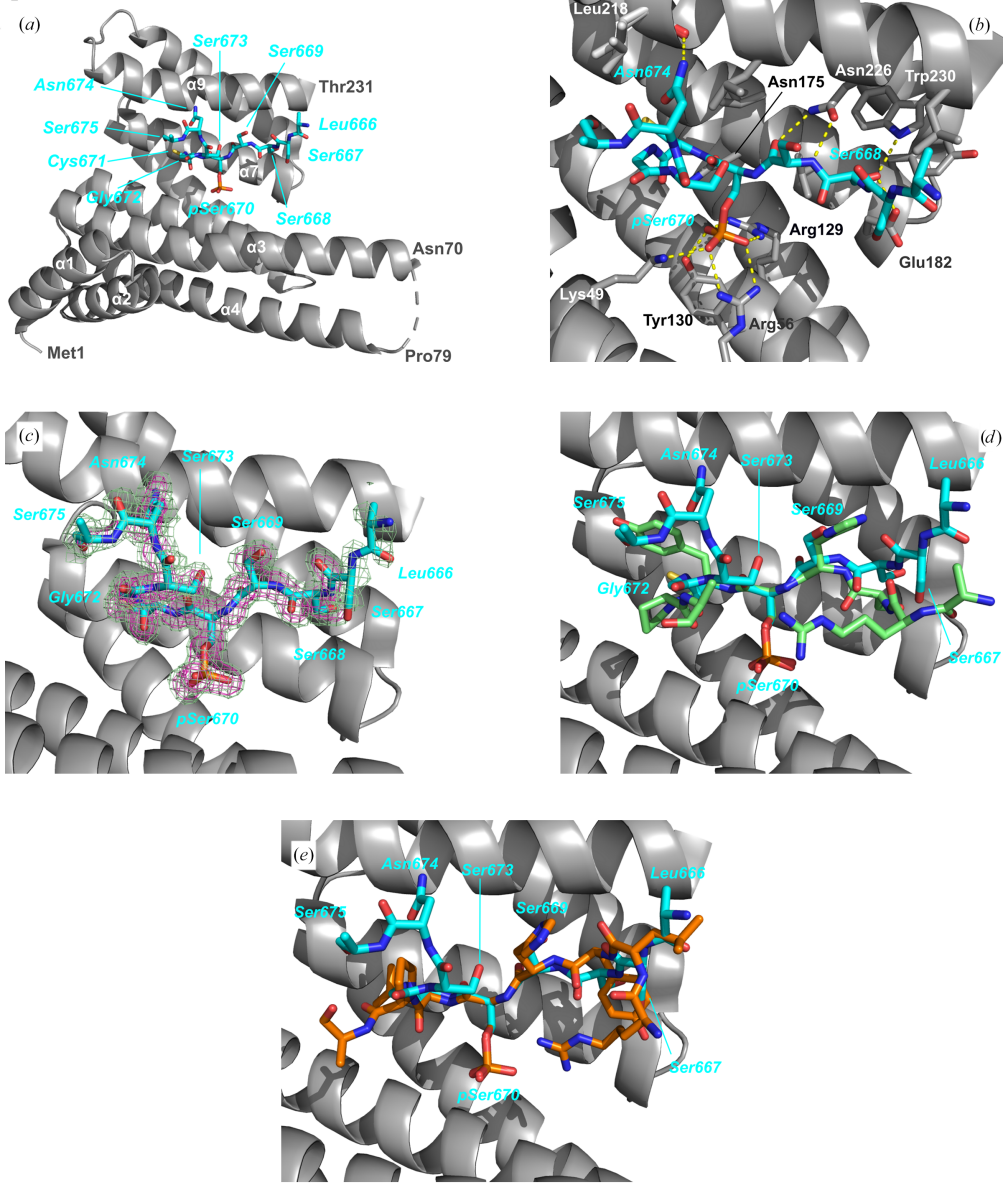
Crystal structure of the 14-3-3σ–Sirt1^pS670^ complex, (*a*) Content of the asymmetric unit showing a 14-3-3σ monomer with bound Sirt1 peptide and missing the loop Asn70–Pro79. The normal physiological 14-3-3 dimer is formed by a symmetry-related molecule (see Supplementary Fig. S1). (*b*) Sirt1 peptide binding site with hydrogen-bonding network. (*c*) OMIT (by omitting peptide atoms) electron densities of Sirt1 peptide contoured at 2σ (green) and at 4σ (magenta). (*d*, *e*) Structure superposition of Sirt1 peptide with (*d*) 14-3-3 binding mode 1 (PDB entry 1qjb) and (*e*) mode 2 (PDB entry 1qja) peptides by fitting the pSer residue using the *Coot* ‘superpose ligand’ function. C-atom coloring: Sirt1 peptide, cyan; mode 1 peptide, green; mode 2 peptide, orange.

**Table 1:** Crystallization of 14-3-3σ–sirtuin peptide complexes

Method	Hanging-drop vapor diffusion
Plate type	VDXm 24-well plate
Temperature (°C)	4
Protein/peptide concentration (m*M*)	1 or 1.5
Buffer composition of protein solution	20 m*M* HEPES–NaOH pH 7.5, 2 m*M* MgCl_2_, 1 m*M* TCEP
Composition of reservoir solution	0.1 *M* HEPES–NaOH pH 7.5, 0.2 *M* CaCl_2_, 26%(*v*/*v*) PEG 400, 5%(*v*/*v*) glycerol, 1 m*M* TCEP
Volume and ratio of drop	1 µl, 50/50% protein/reservoir
Volume of reservoir (µl)	200
Composition of cryoprotectant	Same as reservoir solution
Drop setting	Manual
Seeding	No

**Table 2:** Data collection and processing Values in parentheses are for the highest resolution shell.

	14-3-3σ-ΔC–Sirt3^pS103^	14-3-3σ-ΔC–Sirt1^pS670^
PDB code	8anc	8anb
Diffraction source	BESSY BL14.1	BESSY BL14.1
Wavelength (Å)	0.918409	0.918409
Temperature (K)	100	100
Detector	PILATUS 6M	PILATUS 6M
Space group	*C*222_1_	*C*222_1_
*a*, *b*, *c* (Å)	82.60, 112.38, 62.69	81.01, 95.55, 79.94
Resolution range (Å)	50–1.11 (1.18–1.11)	50.0–1.64 (1.74–1.64)
Total No. of reflections	390768 (50403)	139781 (21387)
No. of unique reflections	111534 (16525)	38278 (6068)
Completeness (%)	97.6 (90.2)	99.6 (98.6)
〈*I*/σ(*I*)〉	13.21 (2.16)	18.32 (4.32)
CC_1/2_	99.9 (73.5)	99.9 (91.0)
*R*_meas_ (%)	6.1 (62.7)	5.0 (34.9)
Overall *B* factor from Wilson plot (Å^2^)	12.916	26.983

**Table 3:** Structure refinement Values in parentheses are for the highest resolution shell.

	14-3-3σ-ΔC–Sirt3^pS103^	14-3-3σ-ΔC–Sirt1^pS670^
Resolution range (Å)	45.68–1.11 (1.14–1.11)	48.89–1.64 (1.682–1.640)
Completeness (%)	87.9 (40.0)	99.6 (97.5)
No. of reflections		
Working set	95338 (3189)	36279 (2588)
Test set	5111 (171)	1943 (122)
Final *R*_cryst_	12.8 (35.2)	17.6 (24.6)
Final *R*_free_	14.5 (37.4)	20.2 (28.5)
No. of non-H atoms		
Total	2307	2147
Protein	1826	1760
Peptide	67	62
Ions	6	2
Waters	408	323
R.m.s. deviations from ideality		
Bonds (Å)	0.020/0.012	0.014/0.012
Angles (°)	1.73/1.65	1.63/1.64
Average *B* factors (Å^2^)		
Protein	15.4	23.6
Peptide	28.6	35.3
Ions	20.1	31.5
Waters	31.1	33.9
Ramachandran plot		
Favored regions (%)	99.6	99.6
Outliers (%)	0.4	0.0
Unmodelled/incomplete residues (%)	0.0	0.4
