# Supplementary material for: Potential 14-3-3 binding sites in sirtuins reveal extended phosphosite-recognition modes
Source: Acta Crystallogr F Struct Biol Commun. 2026 Jan 1;82(Pt 1):32–40. doi: 10.1107/S2053230X25010908 (PMC12809495; doi:10.1107/S2053230X25010908)
Supplement: Supplementary file 1 [file f-82-00032-sup1.pdf]

STRUCTURAL BIOLOGY
COMMUNICATIONS

Volume 82 (2026)

Supporting information for article:

Potential 14-3-3 binding sites in sirtuins reveal extended phosphosite-recognition modes

Michael Weyand, Laura Quast and Clemens Steegborn

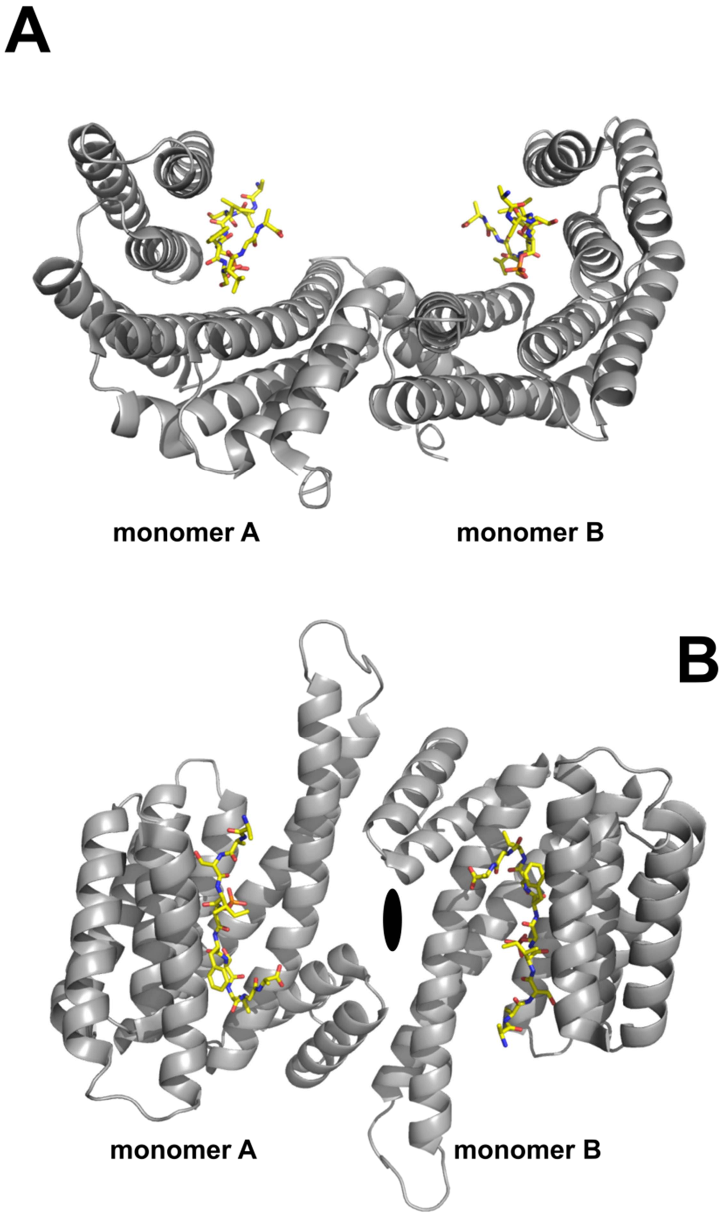
A
monomer A
monomer B
B
monomer A
monomer B

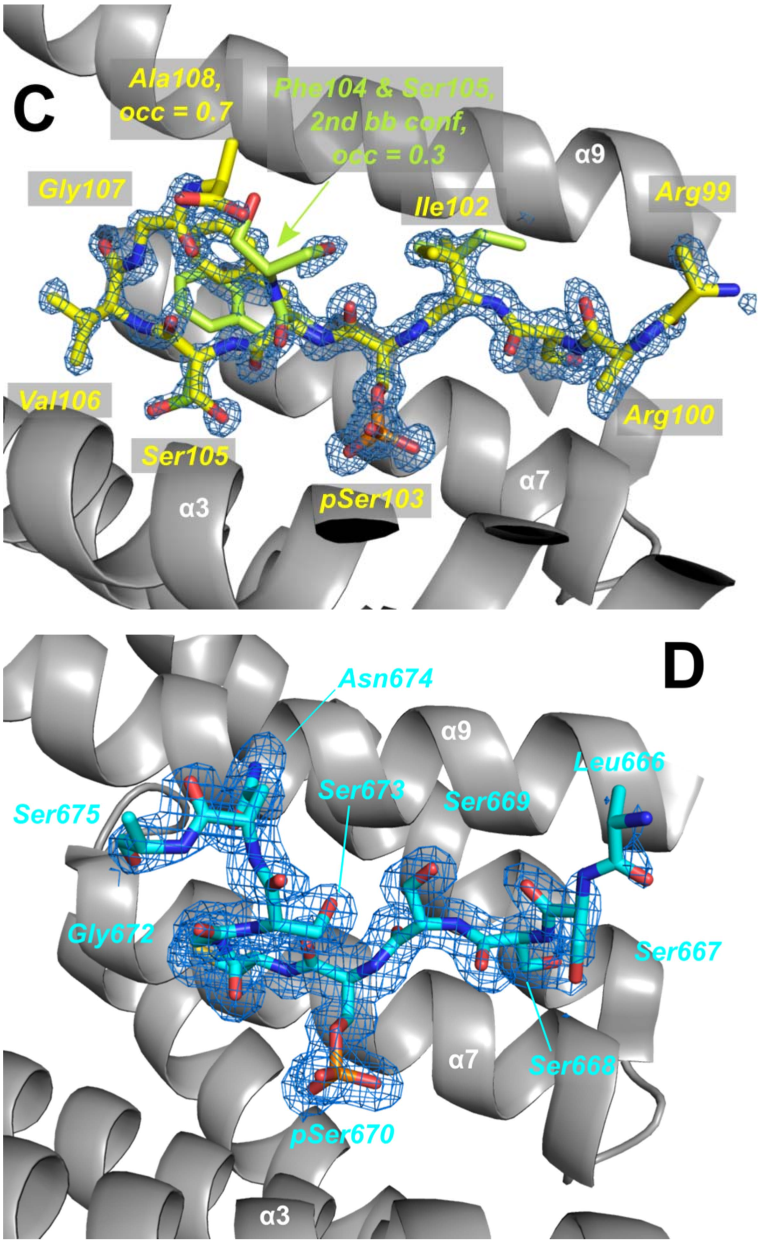

Figure S1 Typical W-shape domain topology of physiological 14-3-3σ homodimer: (A) side view (B) top view with bound sirtuin peptide. Monomer B is a symmetry related molecule within the SG C222(1) packing. Final $2F_o$-F_c electron density for the bound $Sirt3^{pS103}$ (C, PDB 8ANC) and $Sirt1^{pS670}$ (D, PDB 8ANB) endecamers contoured at 1σ.
